# Viral Phylodynamics

**DOI:** 10.1371/journal.pcbi.1002947

**Published:** 2013-03-21

**Authors:** Erik M. Volz, Katia Koelle, Trevor Bedford

**Affiliations:** 1 Department of Epidemiology, University of Michigan, Ann Arbor, Michigan, United States of America; 2 Department of Biology, Duke University, Durham, North Carolina, United States of America; 3 Fogarty International Center, National Institutes of Health, Bethesda, Maryland, United States of America; 4 Institute of Evolutionary Biology, University of Edinburgh, Edinburgh, United Kingdom; University of Toronto, Canada

## Abstract

**Viral phylodynamics** is defined as the study of how epidemiological, immunological, and evolutionary processes act and potentially interact to shape viral phylogenies. Since the coining of the term in 2004, research on viral phylodynamics has focused on transmission dynamics in an effort to shed light on how these dynamics impact viral genetic variation. Transmission dynamics can be considered at the level of cells within an infected host, individual hosts within a population, or entire populations of hosts. Many viruses, especially RNA viruses, rapidly accumulate genetic variation because of short generation times and high mutation rates. Patterns of viral genetic variation are therefore heavily influenced by how quickly transmission occurs and by which entities transmit to one another. Patterns of viral genetic variation will also be affected by selection acting on viral phenotypes. Although viruses can differ with respect to many phenotypes, phylodynamic studies have to date tended to focus on a limited number of viral phenotypes. These include virulence phenotypes, phenotypes associated with viral transmissibility, cell or tissue tropism phenotypes, and antigenic phenotypes that can facilitate escape from host immunity. Due to the impact that transmission dynamics and selection can have on viral genetic variation, viral phylogenies can therefore be used to investigate important epidemiological, immunological, and evolutionary processes, such as epidemic spread [2], spatio-temporal dynamics including metapopulation dynamics [3], zoonotic transmission, tissue tropism [4], and antigenic drift [5]. The quantitative investigation of these processes through the consideration of viral phylogenies is the central aim of viral phylodynamics.

This is a “Topic Page” article for *PLOS Computational Biology*.

## Sources of Phylodynamic Variation

In coining the term *phylodynamics*, Grenfell and coauthors [1] postulated that viral phylogenies “… are determined by a combination of immune selection, changes in viral population size, and spatial dynamics.” Their study showcased three features of viral phylogenies, which may serve as rules of thumb for identifying important epidemiological, immunological, and evolutionary processes influencing patterns of viral genetic variation.

### The Relative Lengths of Internal Versus External Branches Will Be Affected by Changes in Viral Population Size over Time (see Figure 1) [1]

**Figure 1:**
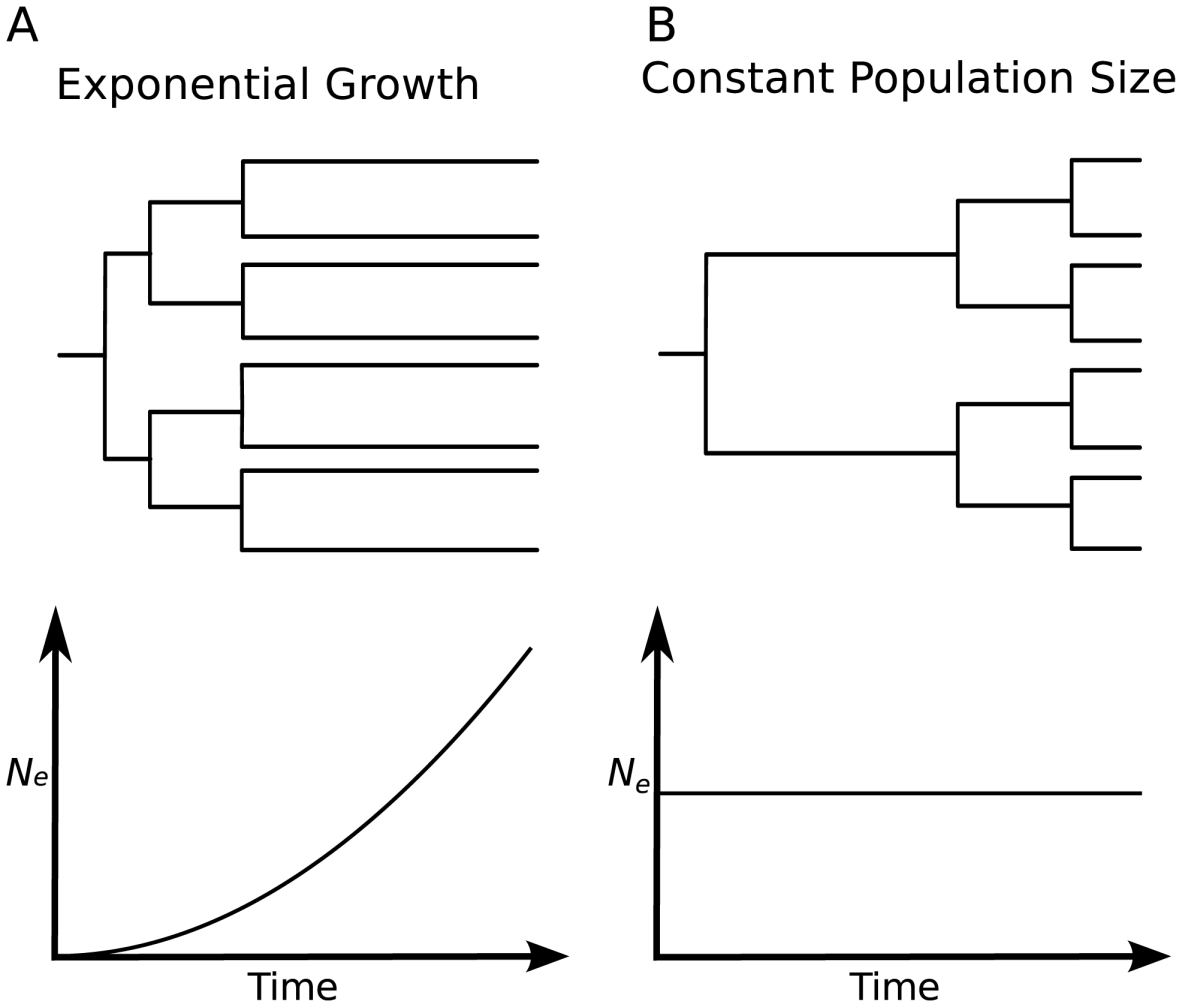
Idealized caricatures of virus phylogenies that show the effects of changes in viral population size.

Rapid expansion of a virus in a population will be reflected by a “star-like” tree, in which external branches are long relative to internal branches. Star-like trees arise because viruses are more likely to share a recent common ancestor when the population is small, and a growing population has an increasingly smaller population size towards the past. Compared to a phylogeny of an expanding virus, a phylogeny of a viral population that stays constant in size will have external branches that are shorter relative to branches on the interior of the tree. The phylogeny of HIV provides a good example of a star-like tree, as the prevalence of HIV infection rose rapidly throughout the 1980s (caricatured by Figure 1A). The phylogeny of hepatitis B virus (caricatured by Figure 1B) instead reflects a viral population that has remained roughly constant in size. Similarly, trees reconstructed from viral sequences isolated from chronically infected individuals can be used to gauge changes in viral population sizes within a host.

### The Clustering of Taxa on a Viral Phylogeny Will Be Affected by Host Population Structure (See Figure 2) [1]

**Figure 2:**
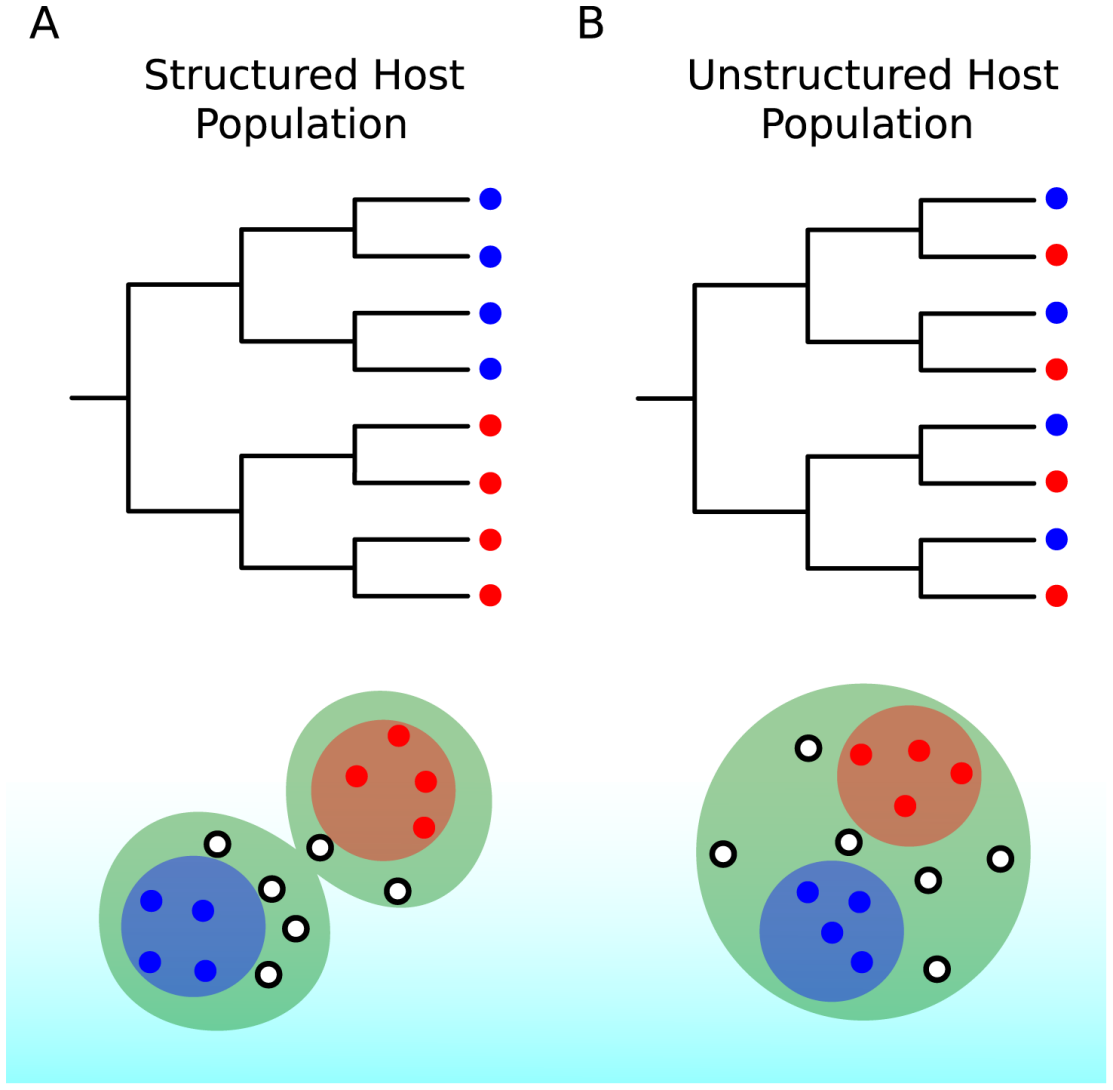
Idealized caricatures of virus phylogenies that show the effects of population structure. Red and blue circles represent spatial locations from which viral samples were isolated.

Viruses within similar hosts, such as hosts that reside in the same geographic region, are expected to be more closely related genetically if transmission occurs more commonly between them. The phylogenies of measles and rabies virus (caricatured by Figure 2A) illustrate viruses with strong spatial structure. These phylogenies stand in contrast to the phylogeny of human influenza, which does not appear to exhibit strong spatial structure over extended periods of time. Clustering of taxa, when it occurs, is not necessarily observed at all scales, and a population that appears structured at some scale may appear panmictic at another scale, for example at a smaller spatial scale. While spatial structure is the most commonly observed population structure in phylodynamic analyses, viruses may also have nonrandom admixture by attributes such as the age, race, and risk behavior [6]. This is because viral transmission can preferentially occur between hosts sharing any of these attributes.

### Tree Balance Will Be Affected by Selection, Most Notably Immune Escape (See Figure 3) [1]

**Figure 3:**
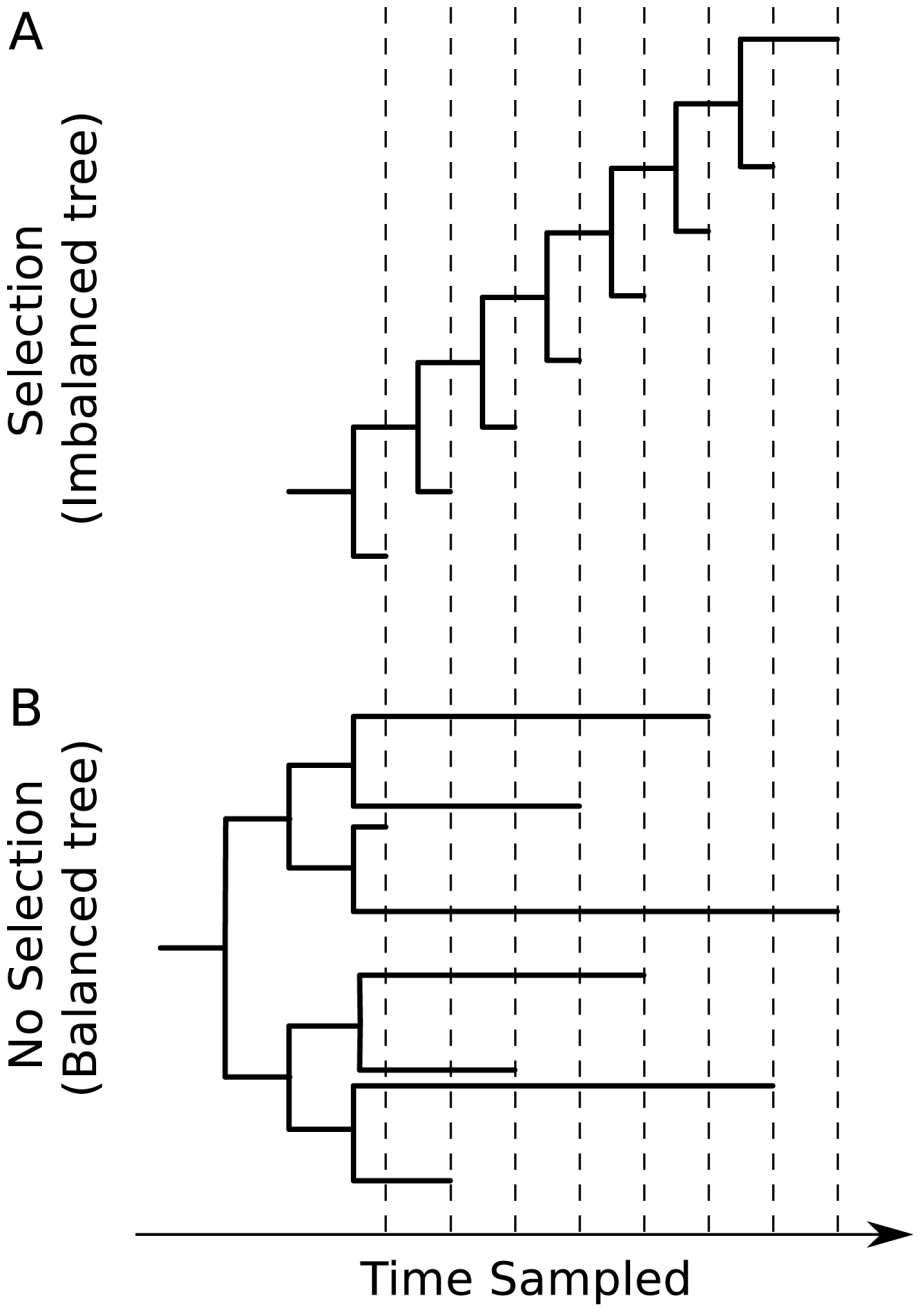
Idealized caricatures of virus phylogenies that show the effects of immune escape.

The effect of directional selection on the shape of a viral phylogeny is exemplified by contrasting the trees of influenza virus and HIV's surface proteins. The ladder-like phylogeny of influenza virus A/H3N2's hemagglutinin protein bears the hallmarks of strong directional selection, driven by immune escape (caricatured by Figure 3A). In contrast, a more balanced phylogeny may occur when a virus is not subject to strong immune selection or other source of directional selection. An example of this is the phylogeny of HIV's envelope protein inferred from sequences isolated from different individuals in a population (caricatured by Figure 3B). Interestingly, phylogenies of HIV's envelope protein from chronically infected hosts resemble influenza's ladder-like tree (caricatured by Figure 3A). This highlights that the processes affecting viral genetic variation can differ across scales. Indeed, contrasting patterns of viral genetic variation within and between hosts has been an active topic in phylodynamic research since the field's inception [1].

Although these three phylogenetic features are useful rules of thumb to identify epidemiological, immunological, and evolutionary processes that might be impacting viral genetic variation, there is growing recognition that the mapping between process and phylogenetic pattern can be many-to-one. For instance, although ladder-like trees such as the one shown in Figure 3A could reflect the presence of directional selection, ladder-like trees could also reflect sequential genetic bottlenecks that might occur with rapid spatial spread, as in the case of rabies virus [7]. Because of this many-to-one mapping between process and phylogenetic pattern, research in the field of viral phylodynamics has sought to develop and apply quantitative methods to effectively infer process from reconstructed viral phylogenies (see Methods). The consideration of other data sources (e.g., incidence patterns) may aid in distinguishing between competing phylodynamic hypotheses. Combining disparate sources of data for phylodynamic analysis remains a major challenge in the field and is an active area of research.

## Applications

### Viral Origins

Phylodynamic models may aid in dating epidemic and pandemic origins. The rapid rate of evolution in viruses allows molecular clock models to be estimated from genetic sequences, thus providing a per-year rate of evolution of the virus. With the rate of evolution measured in real units of time, it is possible to infer the date of the most recent common ancestor (MRCA) for a set of viral sequences. The age of the MRCA of these isolates is a lower bound; the common ancestor of the entire virus population must have existed earlier than the MRCA of the virus sample. In April 2009, genetic analysis of 11 sequences of swine-origin H1N1 influenza suggested that the common ancestor existed at or before 12 January 2009 [8]. This finding aided in making an early estimate of the basic reproduction number *R*_0_ of the pandemic. Similarly, genetic analysis of sequences isolated from within an individual can be used to determine the individual's infection time [9].

### Viral Spread

Phylodynamic models may provide insight into epidemiological parameters that are difficult to assess through traditional surveillance means. For example, assessment of *R*_0_ from surveillance data requires careful control of the variation of the reporting rate and the intensity of surveillance. Inferring the demographic history of the virus population from genetic data may help to avoid these difficulties and can provide a separate avenue for inference of *R*_0_ [2]. Such approaches have been used to estimate *R*_0_ in hepatitis C virus [10] and HIV [2]. Additionally, differential transmission between groups, be they geographic-, age-, or risk-related, is very difficult to assess from surveillance data alone. Phylogeographic models have the possibility of more directly revealing these otherwise hidden transmission patterns [11]. Phylodynamic approaches have mapped the geographic movement of the human influenza virus [3] and quantified the epidemic spread of rabies virus in North American raccoons [12], [13]. However, nonrepresentative sampling may bias inferences of both *R*_0_ [14] and migration patterns [3]. Phylodynamic approaches have also been used to better understand viral transmission dynamics and spread within infected hosts. For example, phylodynamic studies have been used to infer the rate of viral growth within infected hosts and to argue for the occurrence of viral compartmentalization in hepatitis C infection [4].

### Viral Control Efforts

Phylodynamic approaches can also be useful in ascertaining the effectiveness of viral control efforts, particularly for diseases with low reporting rates. For example, the genetic diversity of the DNA-based hepatitis B virus declined in the Netherlands in the late 1990s, following the initiation of a vaccination program [15]. This correlation was used to argue that vaccination was effective at reducing the prevalence of infection, although alternative explanations are possible [16].

Viral control efforts can also impact the rate at which virus populations evolve, thereby influencing phylogenetic patterns. Phylodynamic approaches that quantify how evolutionary rates change over time can therefore provide insight into the effectiveness of control strategies. For example, an application to HIV sequences within infected hosts showed that viral substitution rates dropped to effectively zero following the initiation of antiretroviral drug therapy [17]. This decrease in substitution rates was interpreted as an effective cessation of viral replication following the commencement of treatment, and would be expected to lead to lower viral loads. This finding is especially encouraging because lower substitution rates are associated with slower progression to AIDS in treatment-naive patients [18].

Antiviral treatment also creates selective pressure for the evolution of drug resistance in virus populations, and can thereby affect patterns of genetic diversity. Commonly, there is a fitness trade-off between faster replication of susceptible strains in the absence of antiviral treatment and faster replication of resistant strains in the presence of antivirals [19]. Thus, ascertaining the level of antiviral pressure necessary to shift evolutionary outcomes is of public health importance. Phylodynamic approaches have been used to examine the spread of Oseltamivir resistance in influenza A/H1N1 [20].

## Methods

Most often, the goal of phylodynamic analyses is to make inferences of epidemiological processes from viral phylogenies. Thus, most phylodynamic analyses begin with the reconstruction of a phylogenetic tree. Genetic sequences are often sampled at multiple time points, which allows the estimation of substitution rates and the time of the MRCA using a molecular clock model [21]. For viruses, Bayesian phylogenetic methods are popular because of the ability to fit complex demographic scenarios while integrating out phylogenetic uncertainty [22], [23].

Traditional evolutionary approaches directly utilize methods from computational phylogenetics and population genetics to assess hypotheses of selection and population structure without direct regard for epidemiological models. For example,

- the magnitude of selection can be measured by comparing the rate of nonsynonymous substitution to the rate of synonymous substitution (dN/dS);
- the population structure of the host population may be examined by calculation of F-statistics; and
- hypotheses concerning panmixis and selective neutrality of the virus may be tested with statistics such as Tajimas D.

However, such analyses were not designed with epidemiological inference in mind and it may be difficult to extrapolate from standard statistics to desired epidemiological quantities.

In an effort to bridge the gap between traditional evolutionary approaches and epidemiological models, several analytical methods have been developed to specifically address problems related to phylodynamics. These methods are based on coalescent theory, birth-death models [24], and simulation, and are used to more directly relate epidemiological parameters to observed viral sequences.

### Coalescent Theory and Phylodynamics

#### Effective population size

The coalescent is a mathematical model that describes the ancestry of a sample of nonrecombining gene copies. In modeling the coalescent process, time is usually considered to flow backwards from the present. In a selectively neutral population of constant size N and nonoverlapping generations (the Wright Fisher model), the expected time for a sample of two gene copies to *coalesce* (i.e., find a common ancestor) is N generations. More generally, the waiting time for two members of a sample of *n* gene copies to share a common ancestor is exponentially distributed, with rate
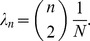



**Figure 4:**
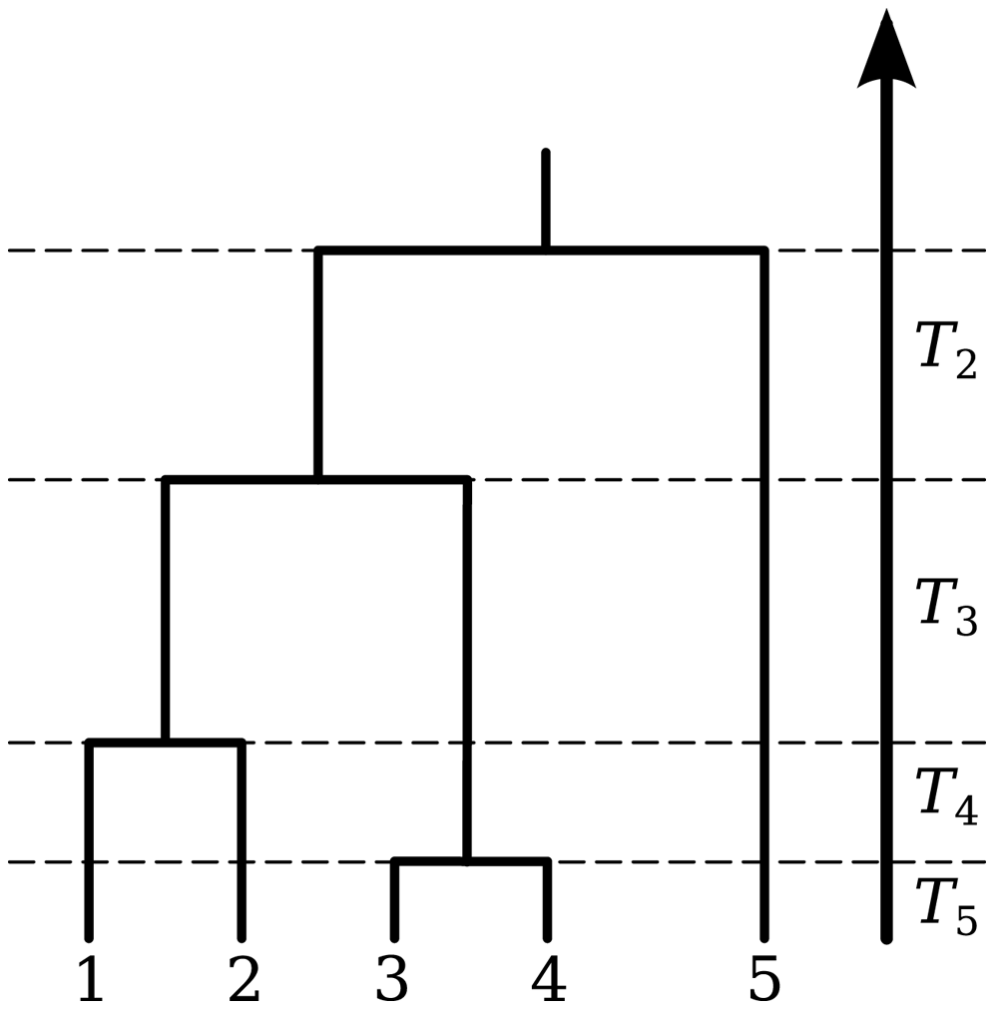
A gene genealogy illustrating internode intervals.

This time interval is labeled *T_n_*, and at its end there are *n*−1 extant lineages remaining (see Figure 4). These remaining lineages will coalesce at the rate 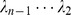 after intervals 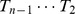. This process can be simulated by drawing exponential random variables with rates 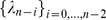 until there is only a single lineage remaining (the MRCA of the sample). In the absence of selection and population structure, the tree topology may be simulated by picking two lineages uniformly at random after each coalescent interval *T_i_*.

The expected waiting time to find the MRCA of the sample is the sum of the expected values of the internode intervals,
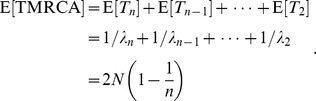



Two corollaries are:

- The time to the MRCA (TMRCA) of a sample is not unbounded in the sample size, 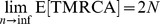.
- Few samples are required for the expected TMRCA of the sample to be close to the theoretical upper bound, as the difference is *O*(1/*n*).

Consequently, the TMRCA estimated from a relatively small sample of viral genetic sequences is an asymptotically unbiased estimate for the time that the viral population was founded in the host population.

For example, Robbins et al. [25] estimated the TMRCA for 74 HIV-1 subtype-B genetic sequences collected in North America to be 1968. Assuming a constant population size, we expect the time back to 1968 to represent 1−1/74 = 99% of the TMRCA of the North American virus population.

If the population size N(*t*) changes over time, the coalescent rate *λ_n_*(*t*) will also be a function of time. Donnelley and Tavaré [26] derived this rate for a time-varying population size under the assumption of constant birth rates:
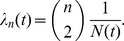



Because all topologies are equally likely under the neutral coalescent, this model will have the same properties as the constant-size coalescent under a rescaling of the time variable:
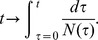



Very early in an epidemic, the virus population may be growing exponentially at rate *r*, so that *t* units of time in the past, the population will have size *N*(*t*) = *N*_0_ *e* ^−*rt*^. In this case, the rate of coalescence becomes
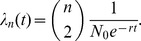



This rate is small close to when the sample was collected (*t* = 0), so that external branches (those without descendants) of a gene genealogy will tend to be long relative to those close to the root of the tree. This is why rapidly growing populations yield trees as depicted in Figure 1A.

If the rate of exponential growth is estimated from a gene genealogy, it may be combined with knowledge of the duration of infection or the serial interval *D* for a particular pathogen to estimate the basic reproduction number, *R*_0_. The two may be linked by the following equation [27]:
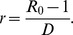



For example, Fraser et al. [8] generated one of the first estimates of *R*_0_ for pandemic H1N1 influenza in 2009 by using a coalescent-based analysis of 11 hemagglutinin sequences in combination with prior data about the infectious period for influenza.

#### Compartmental models

Infectious disease epidemics are often characterized by highly nonlinear and rapid changes in the number of infected individuals and the effective population size of the virus. In such cases, birth rates are highly variable, which can diminish the correspondence between effective population size and the prevalence of infection [28]. Many mathematical models have been developed in the field of mathematical epidemiology to describe the nonlinear time series of prevalence of infection and the number of susceptible hosts. A well studied example is the Susceptible-Infected-Recovered (SIR) system of differential equations, which describes the fractions of the population *S*(*t*) susceptible, *I*(*t*) infected, and *R*(*t*) recovered as a function of time:
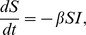


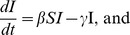


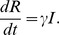
Here, *β* is the per capita rate of transmission to susceptible hosts, and *γ* is the rate at which infected individuals recover, whereupon they are no longer infectious. In this case, the incidence of new infections per unit time is 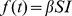, which is analogous to the birth rate in classical population genetics models. Volz et al. [2] proposed that the general formula for the rate of coalescence will be:
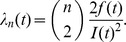



The ratio 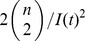 can be understood as arising from the probability that two lineages selected uniformly at random are both ancestral to the sample. This probability is the ratio of the number of ways to pick two lineages without replacement from the set of lineages and from the set of all infections: 
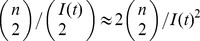
. Coalescent events will occur with this probability at the rate given by the incidence function *f*(*t*).

For the simple SIR model, this yields
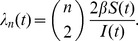



This expression is similar to the Kingman coalescent rate, but is damped by the fraction susceptible *S*(*t*).

Early in an epidemic, *S*(0)≈1, so for the SIR model
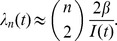



This has the same mathematical form as the rate in the Kingman coalescent, substituting *N_e_* = *I*(*t*)/2*β*. Consequently, estimates of effective population size based on the Kingman coalescent will be proportional to prevalence of infection during the early period of exponential growth of the epidemic [28].

When a disease is no longer exponentially growing but has become endemic, the rate of lineage coalescence can also be derived for the epidemiological model governing the disease's transmission dynamics. This can be done by extending the Wright Fisher model to allow for unequal offspring distributions. With a Wright Fisher generation taking *τ* units of time, the rate of coalescence is given by:
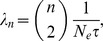
where the effective population size N*_e_* is the population size N divided by the variance of the offspring distribution *σ* ^2^ [29]. The generation time *τ* for an epidemiological model at equilibrium is given by the duration of infection and the population size N is closely related to the equilibrium number of infected individuals. To derive the variance in the offspring distribution *σ* ^2^ for a given epidemiological model, one can imagine that infected individuals can differ from one another in their infectivities, their contact rates, their durations of infection, or in other characteristics relating to their ability to transmit the virus with which they are infected. These differences can be acknowledged by assuming that the basic reproduction number is a random variable *ν* that varies across individuals in the population and that *ν* follows some continuous probability distribution [30]. The mean and variance of these individual basic reproduction numbers, E[*ν*] and Var[*ν*], respectively, can then be used to compute *σ* ^2^. The expression relating these quantities is given by [31]:
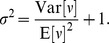



For example, for the SIR model above, modified to include births into the population and deaths out of the population, the population size N is given by the equilibrium number of infected individuals, *I*. The mean basic reproduction number, averaged across all infected individuals, is given by *β*/*γ*, under the assumption that the background mortality rate is negligible compared to the rate of recovery *γ*. The variance in individuals' basic reproduction rates is given by (*β*/*γ*)^2^, because the duration of time individuals remain infected in the SIR model is exponentially distributed. The variance in the offspring distribution *σ* ^2^ is therefore 2. N*_e_* therefore becomes *I*/2 and the rate of coalescence becomes:
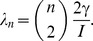



This rate, derived for the SIR model at equilibrium, is equivalent to the rate of coalescence given by the more general formula provided by Volz et al. [2]. Rates of coalescence can similarly be derived for epidemiological models with superspreaders or other transmission heterogeneities, for models with individuals who are exposed but not yet infectious, and for models with variable infectious periods, among others [31]. Given some epidemiological information (such as the duration of infection) and a specification of a mathematical model, viral phylogenies can therefore be used to estimate epidemiological parameters that might otherwise be difficult to quantify.

### Phylogeography

At the most basic level, the presence of geographic population structure can be revealed by comparing the genetic relatedness of viral isolates to geographic relatedness. A basic question is whether geographic character labels are more clustered on a phylogeny than expected under a simple nonstructured model (see Figure 3). This question can be answered by counting the number of geographic transitions on the phylogeny via parsimony, maximum likelihood, or through Bayesian inference. If population structure exists, then there will be fewer geographic transitions on the phylogeny than expected in a panmictic model [32]. This hypothesis can be tested by randomly scrambling the character labels on the tips of the phylogeny and counting the number of geographic transitions present in the scrambled data. By repeatedly scrambling the data and calculating transition counts, a null distribution can be constructed and a p-value computed by comparing the observed transition counts to this null distribution [32].

Beyond the presence or absence of population structure, phylodynamic methods can be used to infer the rates of movement of viral lineages between geographic locations and reconstruct the geographic locations of ancestral lineages. Here, geographic location is treated as a phylogenetic character state, similar in spirit to “A,” “T,” “G,” and “C,” so that geographic location is encoded as a substitution model. The same phylogenetic machinery that is used to infer models of DNA evolution can thus be used to infer geographic transition matrices [33]. The end result is a rate, measured in terms of years or in terms of nucleotide substitutions per site, that a lineage in one region moves to another region over the course of the phylogenetic tree. In a geographic transmission network, some regions may mix more readily and other regions may be more isolated. Additionally, some transmission connections may be asymmetric, so that the rate at which lineages in region “A” move to region “B” may differ from the rate at which lineages in “B” move to “A.” With geographic location thus encoded, ancestral state reconstruction can be used to infer ancestral geographic locations of particular nodes in the phylogeny [33]. These types of approaches can be extended by substituting other attributes for geographic locations. For example, in an application to rabies virus, Streicker and colleagues estimated rates of cross-species transmission by considering host species as the attribute [7].

### Simulation

As discussed above, it is possible to directly infer parameters of simple compartmental epidemiological models, such as SIR models, from sequence data by looking at genealogical patterns. Additionally, general patterns of geographic movement can be inferred from sequence data, but these inferences do not involve an explicit model of transmission dynamics between infected individuals. For more complicated epidemiological models, such as those involving cross-immunity, age structure of host contact rates, seasonality, or multiple host populations with different life history traits, it is often impossible to analytically predict genealogical patterns from epidemiological parameters. As such, the traditional statistical inference machinery will not work with these more complicated models, and in this case, it is common to instead use a forward simulation-based approach.

Simulation-based models require specification of a transmission model for the infection process between infected hosts and susceptible hosts and for the recovery process of infected hosts. Simulation-based models may be compartmental, tracking the numbers of hosts infected and recovered to different viral strains [34], or may be individual-based, tracking the infection state and immune history of every host in the population [5], [35]. Generally, compartmental models offer significant advantages in terms of speed and memory usage, but may be difficult to implement for complex evolutionary or epidemiological scenarios. A forward simulation model may account for geographic population structure or age structure by modulating transmission rates between host individuals of different geographic or age classes. Additionally, seasonality may be incorporated by allowing time of year to influence transmission rate in a stepwise or sinusoidal fashion.

To connect the epidemiological model to viral genealogies requires that multiple viral strains, with different nucleotide or amino acid sequences, exist in the simulation, often denoted *I*_1_, … , *I_n_* for different infected classes. In this case, mutation acts to convert a host in one infected class to another infected class. Over the course of the simulation, viruses mutate and sequences are produced, from which phylogenies may be constructed and analyzed.

For antigenically variable viruses, it becomes crucial to model the risk of transmission from an individual infected with virus strain “A” to an individual who has previously been infected with virus strains “B,” “C,” etc. The level of protection against one strain of virus by a second strain is known as cross-immunity. In addition to risk of infection, cross-immunity may modulate the probability that a host becomes infectious and the duration that a host remains infectious [36]. Often, the degree of cross-immunity between virus strains is assumed to be related to their sequence distance.

In general, in needing to run simulations rather than compute likelihoods, it may be difficult to make fine-scale inferences on epidemiological parameters, and instead, this work usually focuses on broader questions, testing whether overall genealogical patterns are consistent with one epidemiological model or another. Additionally, simulation-based methods are often used to validate inference results, providing test data where the correct answer is known ahead of time. Because computing likelihoods for genealogical data under complex simulation models has proven difficult, an alternative statistical approach called Approximate Bayesian Computation (ABC) is becoming popular in fitting these simulation models to patterns of genetic variation, following successful application of this approach to bacterial diseases [37]–[39]. This is because ABC makes use of easily computable summary statistics to approximate likelihoods, rather than the likelihoods themselves.

## Examples

### Phylodynamics of Influenza

**Figure 5:**
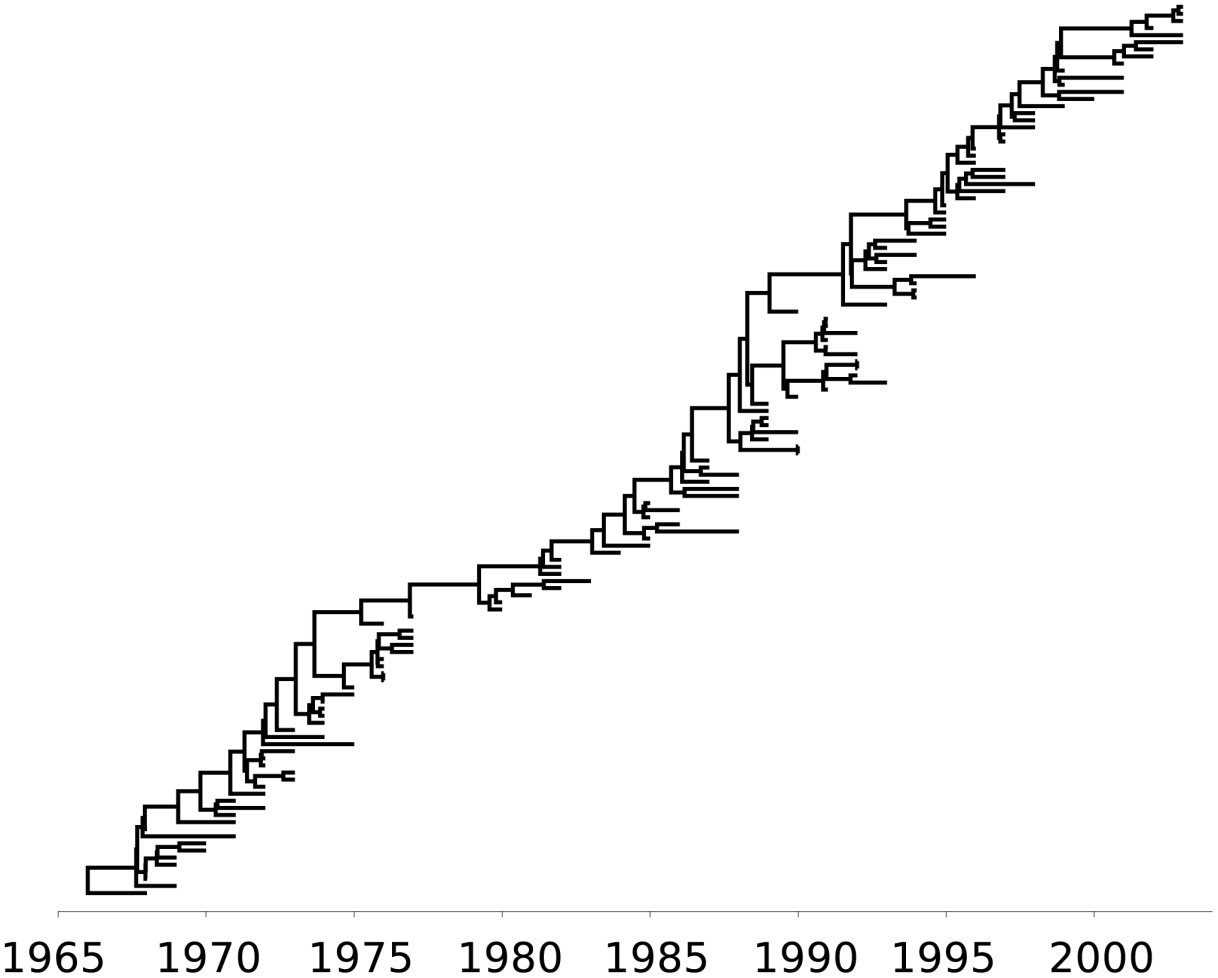
Phylogenetic tree of the HA1 region of the HA gene of influenza A (H3N2) from viruses sampled between 1968 and 2002.

Human influenza is an acute respiratory infection primarily caused by viruses influenza A and influenza B. Influenza A viruses can be further classified into subtypes, such as A/H1N1 and A/H3N2. Here, subtypes are denoted according to their hemagglutinin (H or HA) and neuraminidase (N or NA) genes, which as surface proteins, act as the primary targets for the humoral immune response. Influenza viruses circulate in other species as well, most notably as swine influenza and avian influenza. Through reassortment, genetic sequences from swine and avian influenza occasionally enter the human population. If a particular hemagglutinin or neuraminidase has been circulating outside the human population, then humans will lack immunity to this protein and an influenza pandemic may follow a host switch event, as seen in 1918, 1957, 1968, and 2009. After introduction into the human population, a lineage of influenza generally persists through antigenic drift, in which HA and NA continually accumulate mutations allowing viruses to infect hosts immune to earlier forms of the virus. These lineages of influenza show recurrent seasonal epidemics in temperate regions and less periodic transmission in the tropics. Generally, at each pandemic event, the new form of the virus outcompetes existing lineages [35]. The study of viral phylodynamics in influenza primarily focuses on the continual circulation and evolution of epidemic influenza, rather than on pandemic emergence. Of central interest to the study of viral phylodynamics is the distinctive phylogenetic tree of epidemic influenza A/H3N2, which shows a single predominant trunk lineage that persists through time and side branches that persist for only 1–5 years before going extinct (see Figure 5) [40].

#### Selective pressures

Phylodynamic techniques have provided insight into the relative selective effects of mutations to different sites and different genes across the influenza virus genome. The exposed location of hemagglutinin (HA) suggests that there should exist strong selective pressure for evolution to the specific sites on HA that are recognized by antibodies in the human immune system. These sites are referred to as epitope sites. Phylogenetic analysis of H3N2 influenza has shown that putative epitope sites of the HA protein evolve approximately 3.5 times faster on the trunk of the phylogeny than on side branches (see Figure 5) [41], [42]. This suggests that viruses possessing mutations to these exposed sites benefit from positive selection and are more likely than viruses lacking such mutations to take over the influenza population. Conversely, putative nonepitope sites of the HA protein evolve approximately twice as fast on side branches than on the trunk of the H3 phylogeny [41], [42], indicating that mutations to these sites are selected against and viruses possessing such mutations are less likely to take over the influenza population. Thus, analysis of phylogenetic patterns gives insight into underlying selective forces. A similar analysis combining sites across genes shows that while both HA and NA undergo substantial positive selection, internal genes show low rates of amino acid fixation relative to levels of polymorphism, suggesting an absence of positive selection [43].

Further analysis of HA has shown it to have a very small effective population size relative to the census size of the virus population, as expected for a gene undergoing strong positive selection [44]. However, across the influenza genome, there is surprisingly little variation in effective population size; all genes are nearly equally low [45]. This finding suggests that reassortment between segments occurs slowly enough, relative to the actions of positive selection, that genetic hitchhiking causes beneficial mutations in HA and NA to reduce diversity in linked neutral variation in other segments of the genome.

Influenza A/H1N1 shows a larger effective population size and greater genetic diversity than influenza H3N2 [45], suggesting that H1N1 undergoes less adaptive evolution than H3N2. This hypothesis is supported by empirical patterns of antigenic evolution; there have been nine vaccine updates recommended by the WHO for H1N1 in the interpandemic period between 1978 and 2009, while there have been 20 vaccine updates recommended for H3N2 during this same time period [46]. Additionally, an analysis of patterns of sequence evolution on trunk and side branches suggests that H1N1 undergoes substantially less positive selection than H3N2 [42], [43]. However, the underlying evolutionary or epidemiological cause for this difference between H3N2 and H1N1 remains unclear.

#### Circulation patterns

The extremely rapid turnover of the influenza population means that the rate of geographic spread of influenza lineages must also, to some extent, be rapid. Surveillance data show a clear pattern of strong seasonal epidemics in temperate regions and less periodic epidemics in the tropics [47]. The geographic origin of seasonal epidemics in the Northern and Southern Hemispheres had been a major open question in the field. However, recent work by Rambaut et al. [45] and Russell et al. [48] has shown that temperate epidemics usually emerge from a global reservoir rather than emerging from within the previous season's genetic diversity. This work, and more recent work by Bedford et al. [3] and Bahl et al. [49], has suggested that the global persistence of the influenza population is driven by viruses being passed from epidemic to epidemic, with no individual region in the world showing continual persistence. However, there is considerable debate regarding the particular configuration of the global network of influenza, with one hypothesis suggesting a metapopulation in East and Southeast Asia that continually seeds influenza in the rest of the world [48], and another hypothesis advocating a more global metapopulation in which temperate lineages often return to the tropics at the end of a seasonal epidemic [3], [49].

All of these phylogeographic studies necessarily suffer from limitations in the worldwide sampling of influenza viruses. For example, the relative importance of tropical Africa and India has yet to be uncovered. Additionally, the phylogeographic methods used in these studies (see section on phylogeographic methods) make inferences of the ancestral locations and migration rates on only the samples at hand, rather than on the population in which these samples are embedded. Because of this, study-specific sampling procedures are a concern in extrapolating to population-level inferences. However, through joint epidemiological and evolutionary simulations, Bedford et al. [3] show that their estimates of migration rates appear robust to a large degree of undersampling or oversampling of a particular region. Further methodological progress is required to more fully address these issues.

#### Simulation-based models

Forward simulation-based approaches for addressing how immune selection can shape the phylogeny of influenza A/H3N2's hemagglutinin protein have been actively developed by disease modelers since the early 2000s. These approaches include both compartmental models and agent-based models. One of the first compartmental models for influenza was developed by Gog and Grenfell [34], who simulated the dynamics of many strains with partial cross-immunity to one another. Under a parameterization of long host lifespan and short infectious period, they found that strains would form self-organized sets that would emerge and replace one another. Although the authors did not reconstruct a phylogeny from their simulated results, the dynamics they found were consistent with a ladder-like viral phylogeny exhibiting low strain diversity and rapid lineage turnover.

Later work by Ferguson and colleagues [35] adopted an agent-based approach to better identify the immunological and ecological determinants of influenza evolution. The authors modeled influenza's hemagglutinin as four epitopes, each consisting of three amino acids. They showed that under strain-specific immunity alone (with partial cross-immunity between strains based on their amino acid similarity), the phylogeny of influenza A/H3N2's HA was expected to exhibit “explosive genetic diversity,” a pattern that is inconsistent with empirical data. This led the authors to postulate the existence of a temporary strain-transcending immunity: individuals were immune to reinfection with any other influenza strain for approximately six months following an infection. With this assumption, the agent-based model could reproduce the ladder-like phylogeny of influenza A/H3N2's HA protein.

Work by Koelle and colleagues [5] revisited the dynamics of influenza A/H3N2 evolution following the publication of a paper by Smith and colleagues [50], which showed that the antigenic evolution of the virus occurred in a punctuated manner. The phylodynamic model designed by Koelle and coauthors argued that this pattern reflected a many-to-one genotype-to-phenotype mapping, with the possibility of strains from antigenically distinct clusters of influenza sharing a high degree of genetic similarity. Through incorporating this mapping of viral genotype into viral phenotype (or antigenic cluster) into their model, the authors were able to reproduce the ladder-like phylogeny of influenza's HA protein without generalized strain-transcending immunity. The reproduction of the ladder-like phylogeny resulted from the viral population passing through repeated selective sweeps. These sweeps were driven by herd immunity and acted to constrain viral genetic diversity.

Instead of modeling the genotypes of viral strains, a compartmental simulation model by Gökaydin and colleagues [51] considered influenza evolution at the scale of antigenic clusters (or phenotypes). This model showed that antigenic emergence and replacement could result under certain epidemiological conditions. These antigenic dynamics would be consistent with a ladder-like phylogeny of influenza exhibiting low genetic diversity and continual strain turnover.

In recent work, Bedford and colleagues [52] used an agent-based model to show that evolution in a Euclidean antigenic space can account for the phylogenetic pattern of influenza A/H3N2's HA, as well as the virus's antigenic, epidemiological, and geographic patterns. The model showed the reproduction of influenza's ladder-like phylogeny depended critically on the mutation rate of the virus as well as the immunological distance yielded by each mutation.

#### The phylodynamic diversity of influenza

Although most research on the phylodynamics of influenza has focused on seasonal influenza A/H3N2 in humans, influenza viruses exhibit a wide variety of phylogenetic patterns. Qualitatively similar to the phylogeny of influenza A/H3N2's hemagglutinin protein (see Figure 5), influenza A/H1N1 exhibits a ladder-like phylogeny with relatively low genetic diversity at any point in time and rapid lineage turnover [35]. However, the phylogeny of influenza B's hemagglutinin protein has two circulating lineages: the Yamagata and the Victoria lineage [53]. It is unclear how the population dynamics of influenza B contribute to this evolutionary pattern, although one simulation model has been able to reproduce this phylogenetic pattern with longer infectious periods of the host [54].

Genetic and antigenic variation of influenza is also present across a diverse set of host species. The impact of host population structure can be seen in the evolution of equine influenza A/H3N8: instead of a single trunk with short side-branches, the hemagglutinin of influenza A/H3N8 splits into two geographically distinct lineages, representing American and European viruses [55], [56]. The evolution of these two lineages is thought to have occurred as a consequence of quarantine measures [55]. Additionally, host immune responses are hypothesized to modulate virus evolutionary dynamics. Swine influenza A/H3N2 is known to evolve antigenically at a rate that is six times slower than that of the same virus circulating in humans, although these viruses' rates of genetic evolution are similar [57]. Influenza in aquatic birds is hypothesized to exhibit “evolutionary stasis” [58], although recent phylogenetic work indicates that the rate of evolutionary change in these hosts is similar to those in other hosts, including humans [59]. In these cases, it is thought that short host lifespans prevent the build-up of host immunity necessary to effectively drive antigenic drift.

### Phylodynamics of HIV

#### Origin and spread

The global diversity of HIV-1 group M is shaped by its origins in Central Africa around the turn of the 20th century. The epidemic underwent explosive growth throughout the early 20th century with multiple radiations out of Central Africa. While traditional epidemiological surveillance data are almost nonexistent for the early period of epidemic expansion, phylodynamic analyses based on modern sequence data can be used to estimate when the epidemic began and to estimate the early growth rate. The rapid early growth of HIV-1 in Central Africa is reflected in the star-like phylogenies of the virus (caricatured in Figure 2), with most coalescent events occurring in the distant past. Multiple founder events have given rise to distinct HIV-1 group M subtypes which predominate in different parts of the world. Subtype B is most prevalent in North America and Western Europe, while subtypes A and C, which account for more than half of infections worldwide, are common in Africa [60]. HIV subtypes differ slightly in their transmissibility, virulence, effectiveness of antiretroviral therapy, and pathogenesis [61].

The rate of exponential growth of HIV in Central Africa in the early 20th century preceding the establishment of modern subtypes has been estimated using coalescent approaches. Several estimates based on parametric exponential growth models are shown in Table 1, for different time periods, risk groups, and subtypes. The early spread of HIV-1 has also been characterized using nonparametric (“skyline”) estimates of N*_e_* [62].

**Table 1:** Estimated annual growth rates of *N_e_* for early HIV sub-epidemics.

Growth Rate	Group	Subtype	Risk Group
0.17 [83]	M	NA	Central Africa
0.27 [84]	M	C	Central Africa
0.48 [67]–0.83 [65]	M	B	North America/Eur/Aust, MSM
0.068 [63]	O	NA	Cameroon

The early growth of subtype B in North America was quite high, however the duration of exponential growth was relatively short, with saturation occurring in the mid- and late-1980s [2]. At the opposite extreme, HIV-1 group O, a relatively rare group that is geographically confined to Cameroon and that is mainly spread by heterosexual sex, has grown at a lower rate than either subtype B or C [63].

HIV-1 sequences sampled over a span of five decades have been used with relaxed molecular clock phylogenetic methods to estimate the time of cross-species viral spillover into humans around the early 20th century [64]. The estimated TMRCA for HIV-1 coincides with the appearance of the first densely populated large cities in Central Africa. Similar methods have been used to estimate the time that HIV originated in different parts of the world. The origin of subtype B in North America is estimated to be in the 1960s, where it went undetected until the AIDS epidemic in the 1980s [65]. There is evidence that progenitors of modern subtype B originally colonized the Caribbean before undergoing multiple radiations to North and South America [66]. Subtype C originated around the same time in Africa [67].

#### Contemporary epidemiological dynamics

At shorter time scales and finer geographical scales, HIV phylogenies may reflect epidemiological dynamics related to risk behavior and sexual networks. Very dense sampling of viral sequences within cities over short periods of time has given a detailed picture of HIV transmission patterns in modern epidemics. Sequencing of virus from newly diagnosed patients is now routine in many countries for surveillance of drug resistance mutations, which has yielded large databases of sequence data in those areas. There is evidence that HIV transmission within heterogeneous sexual networks leaves a trace in HIV phylogenies, in particular making phylogenies more imbalanced and concentrating coalescent events on a minority of lineages [68].

By analyzing phylogenies estimated from HIV sequences from men who have sex with men in London, United Kingdom, Lewis et al. found evidence that transmission is highly concentrated in the brief period of primary HIV infection (PHI), which consists of approximately the first 6 months of the infectious period [69]. In a separate analysis, Volz et al. [70] found that simple epidemiological dynamics explain phylogenetic clustering of viruses collected from patients with PHI. Patients who were recently infected were more likely to harbor virus that is phylogenetically close to samples from other recently infected patients. Such clustering is consistent with observations in simulated epidemiological dynamics featuring an early period of intensified transmission during PHI. These results therefore provided further support for Lewis et al.'s findings that HIV transmission occurs frequently from individuals early in their infection.

#### Viral adaptation

**Figure 6:**
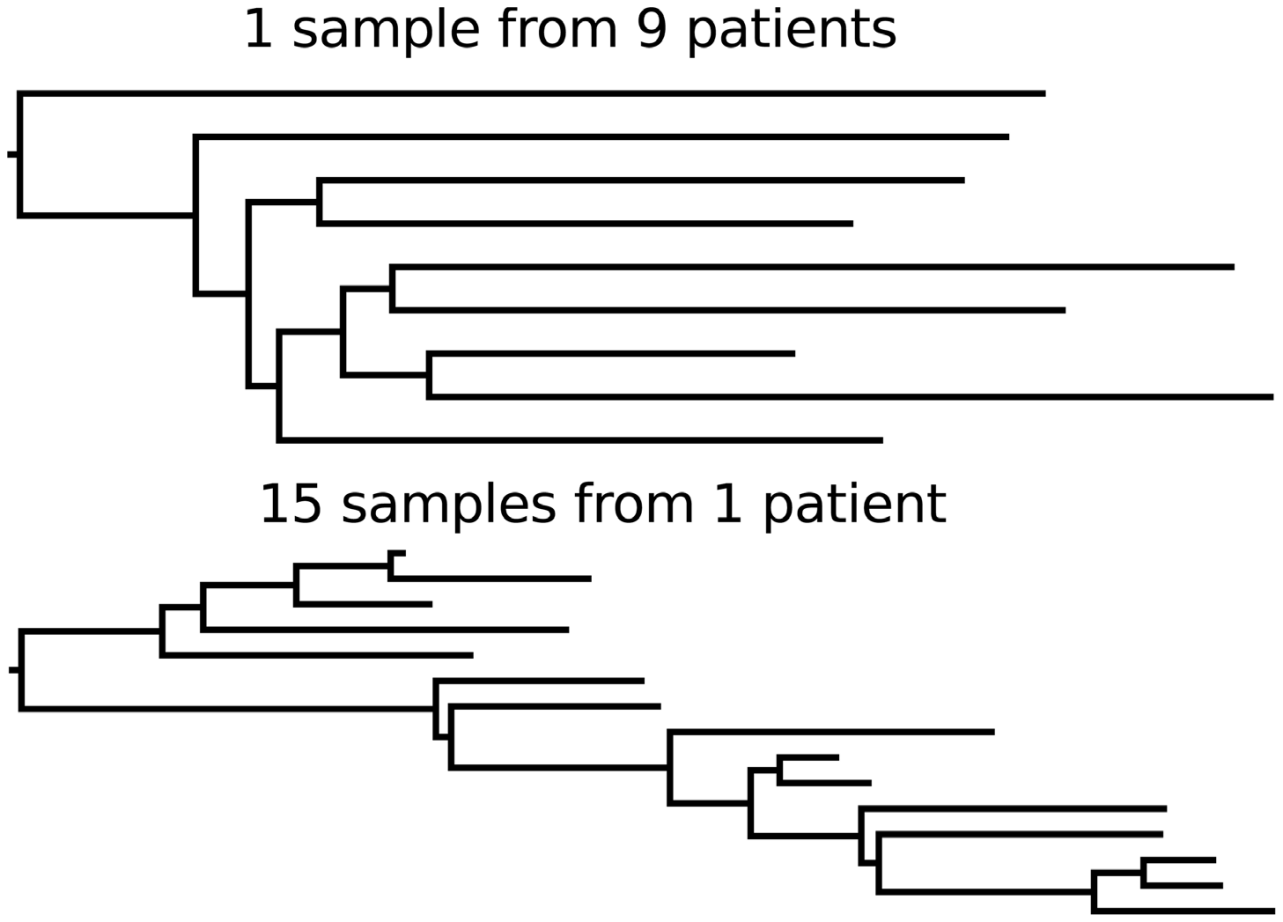
Between-host and within-host HIV phylogenies. Sequences were downloaded from the LANL HIV sequence database (http://www.hiv.lanl.gov/content/sequence/HIV/SI_alignments/set1.html). Neighbor-joining trees were estimated from Alignment1, and the within host tree is based on data from patient 2. Trees were re-rooted using Path-o-gen using known sample dates (http://tree.bio.ed.ac.uk/software/pathogen/).

Purifying immune selection dominates evolution of HIV within hosts, but evolution between hosts is largely decoupled from within-host evolution (see Figure 6) [71]. Immune selection has relatively little influence on HIV phylogenies at the population level for three reasons. First, there is an extreme bottleneck in viral diversity at the time of sexual transmission [72]. Second, transmission tends to occur early in infection before immune selection has had a chance to operate [73]. Finally, the replicative fitness of a viral strain (measured in transmissions per host) is largely extrinsic to virological factors, depending more heavily on behaviors in the host population. These include heterogeneous sexual and drug-use behaviors.

There is some evidence from comparative phylogenetic analysis and epidemic simulations that HIV adapts at the level of the population to maximize transmission potential between hosts [74]. This adaptation is towards intermediate virulence levels, which balances the productive lifetime of the host (time until AIDS) with the transmission probability per act. A useful proxy for virulence is the set-point viral load (SPVL), which is correlated with the time until AIDS [75]. SPVL is the quasi-equilibrium titer of viral particles in the blood during chronic infection. For adaptation towards intermediate virulence to be possible, SPVL needs to be heritable and a trade-off between viral transmissibility and the lifespan of the host needs to exist. SPVL has been shown to be correlated between HIV donor and recipients in transmission pairs [76], thereby providing evidence that SPVL is at least partly heritable. The transmission probability of HIV per sexual act is positively correlated with viral load [77], [78], thereby providing evidence of the trade-off between transmissibility and virulence. It is therefore theoretically possible that HIV evolves to maximize its transmission potential. Epidemiological simulation and comparative phylogenetic studies have shown that adaptation of HIV towards optimum SPVL could be expected over 100–150 years [79]. These results depend on empirical estimates for the transmissibility of HIV and the lifespan of hosts as a function of SPVL.

## Future Directions

Up to this point, phylodynamic approaches have focused almost entirely on RNA viruses, which often have mutation rates on the order of 10^−3^ to 10^−4^ substitutions per site per year [80]. This allows a sample of around 1,000 bases to have power to give a fair degree of confidence in estimating the underlying genealogy connecting sampled viruses. However, other pathogens may have significantly slower rates of evolution. DNA viruses, such as herpes simplex virus, evolve orders of magnitude more slowly [81]. These viruses have commensurately larger genomes. Bacterial pathogens such as pneumococcus and tuberculosis evolve slower still and have even larger genomes. In fact, there exists a very general negative correlation between genome size and mutation rate across observed systems [82]. Because of this, similar amounts of phylogenetic signal are likely to result from sequencing full genomes of RNA viruses, DNA viruses, or bacteria. As sequencing technologies continue to improve, it is becoming increasingly feasible to conduct phylodynamic analyses on the full diversity of pathogenic organisms.

Additionally, improvements in sequencing technologies will allow detailed investigation of within-host evolution, as the full diversity of an infecting quasispecies may be uncovered given enough sequencing effort.

## Supporting Information

Text S1 Version history of the text file.
(XML)

Text S2 Peer reviews and response to reviews. Human-readable versions of the reviews and authors' responses are available as comments on this article.
(XML)
